# The Importance of Networking in Autism Gaze Analysis

**DOI:** 10.1371/journal.pone.0141191

**Published:** 2015-10-23

**Authors:** Quentin Guillon, Mohammad H. Afzali, Bernadette Rogé, Sophie Baduel, Jeanne Kruck, Nouchine Hadjikhani

**Affiliations:** 1 URI Octogone, University Toulouse Jean Jaurès, Toulouse, France; 2 Institut Universitaire de France (IUF), Paris, France; 3 Harvard Medical School / MGH / MIT, Martinos Center for Biomedical Imaging, Charlestown, Massachusetts, United States of America; 4 Gillberg Neuropsychiatry Center, University of Gothenburg, Gothenburg, Sweden; Birkbeck, University of London, UNITED KINGDOM

## Abstract

Visual scanning of faces in individuals with Autism Spectrum Disorder (ASD) has been intensively studied using eye-tracking technology. However, most of studies have relied on the same analytic approach based on the quantification of fixation time, which may have failed to reveal some important features of the scanning strategies employed by individuals with ASD. In the present study, we examined the scanning of faces in a group of 20 preschoolers with ASD and their typically developing (TD) peers, using both classical fixation time approach and a new developed approach based on transition matrices and network analysis. We found between group differences in the eye region in terms of fixation time, with increased right eye fixation time for the ASD group and increased left eye fixation time for the TD group. Our complementary network approach revealed that the left eye might play the role of an anchor in the scanning strategies of TD children but not in that of children with ASD. In ASD, fixation time on the different facial parts was almost exclusively dependent on exploratory activity. Our study highlights the importance of developing innovative measures that bear the potential of revealing new properties of the scanning strategies employed by individuals with ASD.

## Introduction

Autism Spectrum Disorder (ASD), which affects as many as 1 in 68 children, is a behaviorally defined neurodevelopmental disorder characterized by persistent deficits in the socio-communicative domain as well as by the presence of restricted and repetitive patterns of behavior, interests or activities [1,2]. Core symptoms of the socio-communicative domain involve atypical eye contact, difficulties in reading other’s facial expressions and intentions and limited understanding of social emotions. These deficits in spontaneously interpreting facial information may somewhat contribute to the social interaction difficulties experienced by individuals with ASD in real-life situations. Faces convey critical information for social interaction, such as a person’s identity, sex, age, emotions, and intentions, and a failure to process facial information effectively likely limits the ability of individuals with ASD to adequately interact with others [3,4].

Several authors have proposed that atypical face processing skills encountered among individuals with ASD might be linked to abnormal face scanning strategies [5–9]. Among the different methods that have been used to study the scanning patterns of individuals with ASD, eye-tracking technology has received a lot of emphasis since it provides accurate, objective and direct measures of eye movements [10].

Several studies have shown a link between an atypical visual scanning strategy of faces and identity or facial expressions recognition problems [5,7,11–13]. However, the nature of the atypicality is controversial, some studies showing differences between groups in the time spent in the eye region [11,12,14,15], others in the time spent in the mouth [5,16]. Furthermore, an atypical visual scanning of faces is not systematically found in the ASD population [17–21]. In a meta-analysis, including 14 eye-tracking studies on face processing in children with ASD, Papagiannopoulou et al. [22] found that reduced gaze fixation to the eye region of faces was significantly associated with ASD, but not atypical mouth fixation. The authors noted however that this latter result should be considered with caution given the high heterogeneity in the mouth fixation studies. More recently, an atypical lateralization of gaze behavior during visual scanning of faces in ASD has also been documented in several studies. Dundas et al. [23] found that adults with ASD do not show a left-right asymmetry towards the left hemiface in the number of fixation and fixation time. Similar results were found for 11 month-old infants at-risk for ASD [24]. This absence of left-right asymmetry in ASD has also been evidenced for the direction of the first fixation when faces are presented at the central vision [25]. However, Yi et al. [26] found that children with ASD looked significantly longer at the left eye, but shorter at the right eye compared to TD controls. Although this latter study does not specifically test for a left-right asymmetry, the pattern of results suggests a typical lateralization of the scanning behavior of individuals with ASD. Thus, despite the many studies conducted in the field, understanding what characterizes the visual scanning of faces in individuals with ASD is still limited.

The vast majority of eye-tracking studies have taken a similar approach to data analysis, based on the quantification of total fixation time for various facial features. Areas of interest (AOIs) representing facial features (e.g., eyes, nose, mouth, etc.) are defined *a priori*, and the sum of the fixation durations on each of these AOIs is then computed, usually expressed as a percentage of total fixation time on the face. At least two reasons can explain the dominance of this approach: the total fixation time is easily quantifiable and analysis software provided by manufacturers can calculate it directly. Additionally, fixation times enable intuitive interpretation of results since it is thought as reflecting the relative importance (interest) of an AOI in visual scanning for a given individual. The systematic use of this approach has a number of benefits, including facilitating the comparison of results between studies. However, it only partially describes the visual scanning behavior. Fixation time analysis does not take into account the transitions (saccades) between AOIs (i.e. facial features) even though saccading between facial features is an important aspect of visual scanning of faces [27]. Only a handful of studies has focused on this aspect of gaze behavior in ASD [13,28,26,29]. Wilson et al. [13] for example have shown that differences between ASD and TD controls in a task probing memory for faces is better reflected by differences in the amount of saccades between different facial features during encoding rather than the total fixation time for any of these features. In another study, Shic et al. [28] examined exploratory behaviors across the face using the number of transition and the entropy of scanning patterns. They found that exploratory behaviors, especially across the inner facial features, increased with age in TD children but not in children with ASD. These findings suggest that exploratory behaviors across the face differ between ASD and TD individuals, that is not only there are differences in the amount of time spent on certain facial features, but also on the saccading strategies between those facial features.

The present study was conducted to address these two aspects of the visual scanning of faces in a group of preschool children with ASD and age-matched controls. We examined whether children with ASD and TD children look at the main facial features (i.e. left eye, right eye, nose, mouth, outer facial features) differently. First, we employed the traditional fixation time analysis approach to quantify the amount of time spent within each of these facial features. Second, we developed a measure summarizing exploratory behaviors associated with every single facial features independently. We reasoned that one limitation of the measures that have been used so far to investigate exploratory behaviors is that they summarized exploratory behaviors associated with a set of facial features (e.g. between the eyes and mouth region). Our measure quantify the importance of each facial features in visual scanning based on their pattern of transitions with other facial features, thereby allowing the investigation of exploratory behaviors at a local level. To compute this new measure, we borrowed concepts and data processing methods from network analysis [30,31]. The development, formalization and the complete procedure of calculating this measure are presented below.

## Materials and Methods

### Participants

All parents or legal guardians provided their written informed consent to participate in the study in accordance with the principles explained in the Declaration of Helsinki, and all procedures involved in the present study were approved by the Octogone ethics committee.

Twenty children with ASD and 21 typically developing (TD) children participated in this study. No difference between groups was observed for chronological age, *t* (39) = -0,788, *p* = .435 and the sex ratio, χ2 (1, N = 41) = 1.62, *p* = .203. Non-verbal mental age (NVMA) and verbal mental age (VMA) was significantly lower in the ASD group (NVMA: *t* (39) = -2.96, *p* = .005; VMA: *t* (39) = -4.02, *p* < .001). The diagnosis of ASD was established using algorithm cut-offs on the ADOS (module 1 or 2) and the ADI-R and was further confirmed by one expert clinician (BR, JK) based on DSM-V criteria and a review of all available information. To standardize ADOS scores between modules, a severity score based on Gotham, Pickles, & Lord, [32] was also computed. A detailed description of the two groups of participants is provided in Table 1 below:

**Table 1:** Sample characteristics.

	ASD (*n* = 20)		TD (*n* = 21)		
	M	*SD*	M	*SD*	*p*-value
Chronological age (months)	40.7	11.5	43.9	14.3	ns
NVMA^a^ (months)	33.6	12.6	46.1	14.5	.005
VMA^a^ (months)	28.1	13.6	45.1	13.5	< .001
Sex-ratio (boy/girl)	16/4		13/8		ns
ADOS severity score	6.5	1.8	N/A		-

^a^ NVMA (non verbal mental age) and VMA (verbal mental age) obtained from the Mullen Scales of Early Learning.

### Stimuli

Twelve faces from the Ekman POFA collections were used in this study [33]: four faces (2 women) with a negative facial expression (anger), four faces (2 women) with a neutral facial expression, and four faces (2 women) with a positive facial expression (happy). All photographs subtended the same size (about 19° x 20° at a distance of 60 cm), and had a uniform gray background, so that the left and the right side of each photograph did not differ in terms of luminance (cd/m2) and contrast (percentage of ‘gray’ pixels). This allows controlling for an effect of the pictures’ low physical properties on the participants lateral eye movements. Note that we used different posed emotional expressions in order to induce exploratory behaviors across the different facial features.

### Procedure

The stimuli were presented using E-prime 2.0 software (Psychology Software Tools Inc., Pittsburgh, Pennsylvania, USA) on a 17-inch screen (1024x 768 resolution), integrated to a Tobii T120 eye-tracking system (Tobii Inc., Stockholm, Sweden). The average accuracy of this eye-tracking system is in the range of 0.4° to 0.6°. The pupil and corneal reflections were recorded binocularly at a sampling rate of 60 Hz.

Light levels were maintained constant for all the participants during recording, and potential distractors were removed. Participants were placed in a car seat adapted and fixed to a chair with an adjustable height at a distance of about 60 cm from the screen. A five-point calibration was performed with a short video with a sound representing a bouncing ball (subtended from 0.3° to 1.5° of visual angle in diameter). The calibrated area corresponded to where the stimuli were presented on the screen and covered the stimuli dimension. If less than four points per eye were properly calibrated, the calibration procedure was repeated until it was satisfactory (i.e. ≥ 4 focus properly calibrated for both eyes as judged by visual inspection of gaze plots provided by the eye tracker).

Each trial began with a central fixation point (subtended 1.5°x 1.5° of visual angle) representing a spinning top on a gray background. We used an active gaze contingent procedure so that for the stimuli to appear, the child had to look at the spinning top for 300 consecutive ms within a time window of 5 s after its onset. This procedure served two purposes: first, it ensured that all participants looked at the same location on the screen when the stimuli appeared and second, that accuracy remained reliable throughout the recording. The stimuli were presented for 3500 ms in a random order, followed by a gray screen with a sound varying from 500 to 800 ms to retain the attention of the child.

### Data reduction and analyses

Gaze data analysis was performed with Matlab (R2011a) with scripts written specifically for the study. Fixations were identified based on the algorithms provided in Wass et al. [34] using a saccade velocity threshold of 35°/s and a minimum fixation duration threshold of 100 milliseconds. Network analysis were performed with the R packages *igraph* [35] and *qgraph* [36].

#### A priori definition of areas of interest

Five rectangular AOIs were manually defined on each face: a) left eye, b) right eye, c) nose, d) mouth and e) outer facial features. More specifically, the left and right eye AOIs included the eyes, eyebrows and eyelashes, the mouth AOI included mouth lips and teeth, and the nose AOI included the nasal ridge and tip, the nostrils and the philtrum. To account for offsets in the data, specifically when studies are conducted with young children, the area of the internal facial features AOIs was expanded by 1° of visual angle on each side.

#### Approach based on fixation time

For each AOI, the percentage of fixation time was computed by dividing the total fixation time on a specific AOI by the total fixation time for the stimulus and by multiplying the ratio by 100. For each participant, the average percentage of total fixation time on each AOI for all faces was then calculated.

#### Approach based on transition matrices.

**Fig 1:**
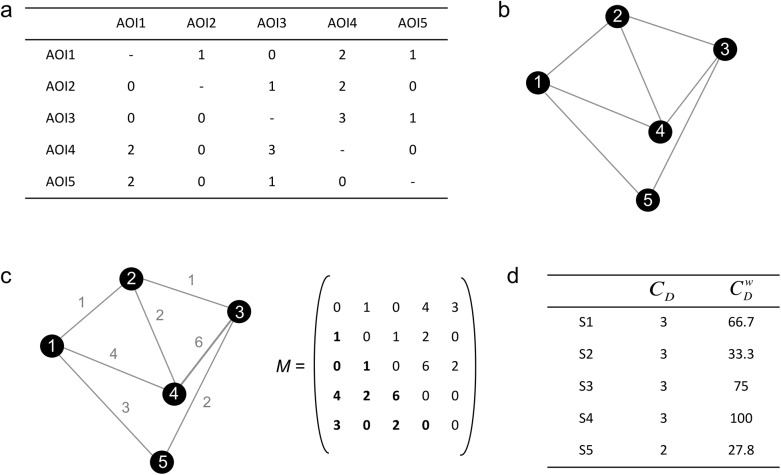
Approach based on transition matrices. a) A fictitious 5 x 5 transition matrix, b) The corresponding undirected network with nodes S1, S2, S3, S4, S5 and links (i.e. transitions) between these nodes, c) The corresponding weighted undirected network with the number of links between pairs of nodes on the left, and weighted adjacency matrix *M* on the right d) Degree centrality for each node in the network.

*Step 1*: *construction of two-dimensional transition matrices*: A transition corresponds to a saccade made from one AOI to another AOI [37]. A two-dimensional transition matrix summarizes all transitions between each possible pairs of AOIs [38] (Fig 1A). Each column and each row of the matrix corresponds to an AOI and the number in each cell represents the number of observed transitions between the two AOIs. By convention, lines indicate the AOI departure and columns indicate the AOI arrival. For example, in the fictitious transition matrix shown in Fig 1A, the participant performs 3 transitions from AOI 3 to AOI 4 and only 1 transition from AOI 3 to AOI 5. Note that saccades performed in one AOI are not considered as transitions and are thus not included in the transition matrix. For the present study, a 5x5 transition matrix, comprising all the transitions made during the exploration of the twelve faces, was first constructed for each participant.

*Step 2*: *construction of adjacency matrices*: A network is characterized by a set of nodes and links connecting pairs of nodes together [30]. Thus, any transition matrix can be regarded as a network where the set of nodes is the set of AOIs and the links correspond to the transitions. A directed network can be defined by a pair *G = (S*, *A)* where

- *S* is a set of nodes
- *A* is a set of node pairs (*S*_*i*_, *S*_*j*_) ∈ *S* ^2^.

To any directed network corresponds an undirected network *G’ = (V*, *A')* such that

- *S* is a set of nodes
- *A'* is a set of nodes pairs verifying

$$\left(S_{i},S_{j}\right)\;\in A^{\prime }\Leftrightarrow \;\left(S_{i},S_{j}\right)\;\in A\;or\;\left(S_{j},S_{i}\right)\;\in A$$

The pairs (*S*_*i*_, *S*_*j*_) ∈ *A*′ are undirected if and only if (*S*_*i*_, *S*_*j*_), is considered equivalent to (*S*_*j*_, *S*_*i*_). For example, Fig 1B, represents an undirected network *G’ = (S*, *A’)*, where *S* = {1, 2, 3, 4, 5} and *A'* = {(1, 2), (1, 4), (1, 5) (2, 3), (2, 4), (3, 4), (3, 5)}.

In a weighted undirected network *G’ = (V*, *A'*, *w)*, each pair (*S*_*i*_, *S*_*j*_) ∈ *A*′ has the weight *w*(*Si*, *Sj*) such that *w* is equal to the number of links between the pair (*S*_*i*_, *S*_*j*_). In the case of transition matrices, the weight of a node pair is thus the number of transitions between a pair of AOIs (Fig 1C).

The concept of adjacency refers to the direct connection between two nodes. In an undirected network *G’ = (V*, *A')*, the node *S*_*i*_ is adjacent to another node *S*_*j*_ if (*S*_*i*_, *S*_*j*_) ∈ *A*′ In Fig 1C, for example, the node S1 is adjacent to the node S2, but not to S3. Similarly, S4 and S3 are adjacent nodes but not S4 and S5. We call an adjacency matrix of a weighted undirected network *G’ = (V*, *A'*, *w)*, the square matrix *M* of size *n x n*, where *n* = |*S*|, and such that each element of *M*, *m*_*ij*_ is equal to *w* if (*i*, *j*) ∈ *A*′, and 0 if (*i*, *j*) ∉ A′. Note that the adjacency matrix of a weighted undirected network is symmetrical about its downward diagonal and therefore only the lower (or higher) triangular component of the matrix should be taken into account to perform all operations on *M* (Fig 1C).

In the present study, a weighted undirected adjacency matrix based on the transitions matrix was generated for each participant with the R packages *igraph* [35] and *qgraph* [36].

*Step 3*: *Calculation of degree centrality*: Centrality of a node is an important concept of network analysis that has been formalized in three different ways: degree, betweenness and closeness [30,31]. In this study, we limited ourselves to degree centrality, which is, according to Freeman [30], “the simplest and perhaps the most intuitively obvious conception” of node centrality. In a weighted undirected network *G’ = (S*, *A'*, *w)*, the degree centrality of a focal node *S*_*i*_, *C*_*D*_(*i*) refers to the number of nodes adjacent to *S*_*i*_, which can be formalized as:

$$C_{D}\left(i\right)=\sum_{j}^{N}M$$

where *i* is the focal node, *j* represents all other nodes, *N* is the total number of nodes in the network and *M*, the *unweighted* oriented adjacency matrix such as the cell *m*_*ij*_ = 1 if *m*_*ij*_ = *w* and 0 if (*i*, *j*) ∉ A′ [30,31]. For example, in Fig 1C, node 1 is directly connected to nodes 2, 4 and 5: therefore its degree centrality is 3. The node 5 is directly connected to nodes 1 and 3: its degree centrality is 2 (Fig 1D).

The degree centrality of a node refers directly to its importance into the network. Applied to transition matrices, the importance of an AOI among others is thus a function of the number of its direct links with these other AOIs. The more a focal AOI has direct links with other AOIs, the more it is important in the network and can be thus considered as a crossroads or anchor of the visual scanning activity.

For each participant, the degree centrality of each AOI was calculated with the R package *qgraph* [36].

The degree centrality as defined above, however, only gives a limited idea of the importance of a node in the network since it simply considers the links on a binary mode (i.e., presence or absence of direct link with the other nodes of the network) and does not take into account the number of these links (i.e., the number of transitions) [31]. This is particularly problematic for small networks, where the probability that all nodes are directly connected to each other is high, and where variability between individuals is likely to be low, as it is the case in the present study.

Barrat, Barthélemy, Pastor-Satorras, & Vespignani [39] proposed to define the degree of a node based on the number of links associated with this node. But, as stressed by Opsahl et al. [31] this solution generates confusion. For example, a degree centrality of 5 might as well result from the fact that the node has 5 direct links with other nodes in the network or 5 links with the same node or a combination of both cases. Opsahl et al. [31] propose a calculation of degree centrality that allows considering these two aspects by applying a tuning parameter according to the relative importance to be given to these two aspects respectively. However, with this solution, the value of the tuning parameter is left to the appreciation of the researcher who can arbitrarily decide to grant more importance to the number of direct links compared to the amount of relationship between nodes, or vice versa.

The computation of degree centrality of a node developed for this study is a data-driven approach, which equally takes into account both the number of nodes that the focal node is connected to and the total number of transition, *(w)*, between the focal node and the other nodes in the network, and which standardizes the measure to make the different nodes degrees comparable among participants. Formally, we have:

$$C_{D}^{w}\left(i\right)=\frac{C_{D}\left(i\right)}{\text{max}\;C_{D}}\;\times \;\frac{w\;\left(i\right)}{\text{max}\;w}\;\times \;100$$

where *C*_*D*_(*i*) represents the degree centrality of the target node (*i*), max *C*_*D*_ is the maximum observed degree for a node in the network, *w*(*i*) represents the number of links to the focal node and max *w*, the maximum number of observed links for a focal node in the network. With this calculation, the value of the degree centrality of a node ranges between 0 and 100 (Fig 1D).

### Statistical Analysis

We performed a series of full factorial ANCOVAs to compare fixation times and centrality degrees between the two groups, taking into account the verbal and non-verbal developmental age and their interaction with group if necessary. All statistical analyses were performed with the R software.

## Results

### Preliminary analysis

In order to ensure that our datasets were comparable between groups, we first compared the total fixation time on stimulus and the total number of transitions. On average, children in the ASD group explored the faces for 2.0 seconds (*SD* = 0.24 seconds), and those from the TD group for 2.0 seconds (*SD* = 0.21 seconds). An independent-samples t-test indicated that total fixation time on stimulus was not significantly different between groups, *t*(39) = -0.07, *p* = .95. The total number of transition per participant in the ASD group was on average 31.7 (*SD* = 12.0) and 35.6 (*SD* = 13.9) in the TD group. The difference was not statistically significant, as indicated by an independent samples t-test, *t*(39) = -0.97, *p* = .34.

### Analysis of fixation time

**Fig 2:**
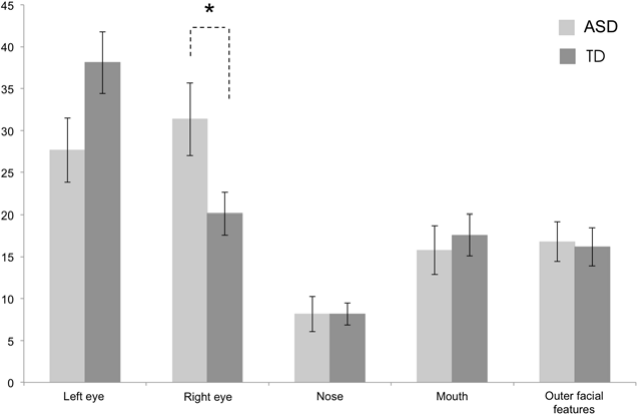
Mean (SE) proportion of fixation time by group for each AOI. * *p* < .05 (uncorrected).

A series of ANCOVAs with group as the between-subjects factor (ASD vs. TD) and verbal and non-verbal developmental age and their interaction with group as covariates was performed for each face AOI fixation time. The results indicate that for the left eye AOI the difference between the two groups was not significant, although marginal significance was observed, *F* (1,35) = 3.56, *p* = .067. A significant difference between the two groups was found for the right eye AOI, *F* (1,35) = 5.28, *p* = .028, partial η2 = 0.13. Children in the ASD group looked longer in the right eye than TD children. No difference between the two groups was found for the mouth AOI, *F* (1,35) = 0.22, *p* = .644, nose AOI, *F* (1.35) = 0.00, *p* = .998 and the outer facial features AOI, *F* (1,35) = 0.04, *p* = .845 (Fig 2). Note that these results are not corrected for multiple comparisons for several reasons. First, given the negative dependency of the data (i.e. increase fixation time to one AOI necessarily leads to a decrease fixation time of the other AOIs) Bonferroni-like correction procedures are not appropriate. Second, considering that for a large proportion of comparisons the null hypothesis is not rejected, applying the alternative False Discovery Rate control procedure from Benjamini and Yekutieli [40] (independent of the dependency properties of the data), would be too conservative, thus increasing false negative, type II error. Taking these points into consideration, we considered that for the present study, decreasing the possibility of type II error was of primary importance compared to familywise error rate.

A more detailed analysis of how visual attention was distributed between the right and the left eye was conducted by computing a left/right eye ratio. A ratio greater than 0.50 indicates a longer fixation time for the left eye. The left/right eye ratio in the ASD group was on average 0.48 (*SD* = 0.26), and 0.64 (*SD* = 0.19) for the TD group. A one-way ANCOVA with group as a between-subject factor (ASD vs. TD) and verbal and non-verbal mental age as covariates revealed a significant difference, *F* (1,37) = 4.95, *p* = .03, partial η2 = 0.12. One sample t-tests (test value = .50, bilateral) revealed an attentional bias for the left eye AOI in the TD group, *t* (20) = 3.37, *p* = .003, *d* = 0,74, but not in the ASD group, *t* (19) = -0.37, *p* = .713.

### Analysis of the degree centrality

A series of ANCOVAs with group as the between-subjects factor (ASD vs. TD) and verbal and non-verbal developmental age and their interaction with group as covariates was performed for each face AOI centrality degree separately. A significant difference for the left eye AOI was found between groups, *F* (1,35) = 4.48, *p* = .041, partial η2 = 0.11. Children in the TD group had a higher degree centrality than children with ASD. No significant differences between groups was found however for the right eye AOI, *F* (1,35) = 0.86, *p* = .361, mouth AOI, *F* (1,35) = 0.02, *p* = .891, nose AOI, *F* (1,35) = 0.51, *p* = .478, and outer facial feature AOI, *F* (1,35) = 0.57, *p* = .454 (Table 2). Like for the fixation time analysis, it should be mentioned that we did not correct for multiple comparisons here for similar reasons.

**Table 2:** Means (SD) of degree centrality by group for each AOI.

	ASD		TD		
	M	*SD*	M	*SD*	*p*-value
Center eye	69.1	33.2	88.1	21.6	.041
Right eye	66.0	33.5	59.9	31.9	ns
Mouth	41.9	30.2	43.2	34.0	ns
Nose	34.9	31.3	28.5	25.4	ns
Outer facial features	44.4	33.1	37.4	25.1	ns

### Analysis of the relationship between degree centrality and fixation time

Both measures depend on the number of fixation associated with an AOI. However, while the fixation time approach considers all fixations to quantify the total fixation time, the approach based on degree centrality only considers fixations that are exploratory in purpose. The degree centrality for an AOI takes also as an input the number of AOIs to which it is directly connected. We thus expected that degree centrality and fixation time would be positively correlated. We performed a series of Pearson correlation analyses between the percentage of fixation time in each AOI and its degree centrality, and as expected, we found that the two measurements are significantly related in both groups (Table 3). The more time spent in an AOI, the higher its degree centrality.

**Table 3:** Correlation coefficients between fixation time and degree centrality by group for each AOI and between group comparison after a Fisher r-to-z transformation.

	ASD		TD		ASD vs. TD	
	*r*	*p*	*r*	*p*	*z*	*p*
Left eye	.841	< .001	.535	< .05	1.856	.03
Right eye	.704	< .001	.885	< .001	- 1.547	.94
Mouth	.826	< .001	.697	< .001	0.928	.18
Nose	.805	< .001	.920	< .001	- 1.409	.92
Outer facial features	.789	< .001	.513	< .05	1.484	.07

In order to determine whether the strength of the relationship between the two variables differed between groups, a comparison of the correlation coefficients for each group and for each AOI was performed after a Fisher r to z transformation. The results indicate that for the left eye AOI, the relationship between fixation time and degree centrality is stronger for ASD than TD (Table 3). No other difference was observed, although a marginal trend was noted for outer facial features.

## Discussion

In this study we examined visual scanning of static faces in young children with ASD. We first conducted a traditional analysis based on fixation time. The results show a difference between ASD and TD in the time spent looking at the left and the right eye AOI. No other difference was found. We then performed an analysis based of transitions between different AOIs. This analysis further showed the importance of the left eye in anchoring facial scanning in TD children, and the lack of this anchoring in ASD children.

In the present study, ASD children spent less time looking at the left eye AOI but more time looking at the right eye AOI than TD children. This is in contradiction with findings from Yi et al, [26,29] who found the opposite pattern of fixation. The reasons for the discrepancy between the current study and Yi et al. [26,29] are not clear. A possible explanation could be differences in task (free viewing vs. face recognition task) and/or presentation time (we collected our fixation data over a fixed duration of 3500ms while Yi et al. collected fixation data combining all different phases of their recognition task, presumably resulting in a longer duration). This discrepancy might also reflect a difference in the ethnicity of the participants. In Yi et al. [26,29], participants were young children of Asian origin, yet several studies have shown differences in the visual scanning of faces between individuals from Western and Asian cultures [41,42]. It is worth mentioning here that our finding should be considered with caution, given increased type I error rate. Thus, replication in futures studies would be desirable.

Nevertheless, and in support to our result, when specifically comparing the left and right eye AOIs, we found that the children in the TD group showed a bias for the left eye, which was not the case for the ASD children, who did not show a bias for any eye. The left eye bias in TD children is consistent with several other studies that have reported that TD children and adults tend to look more into the left than the right hemiface, notably into the left eye region, when faces are presented at central vision [43–46]. This leftward gaze bias has been interpreted in relation to the left visual field superiority and right hemispheric dominance for face processing [47,48]. Although the idea seems counter-intuitive, given that looking to the left puts more of the face in the right visual field, it is usually assumed that the right hemispheric specialization for face processing leads to more interest and exploration of facial features initially presented within the left visual field (i.e. mainly projected to the right hemisphere). Our result of an absence of this left eye bias in ASD children is thus consistent with the idea of an abnormal right hemispheric specialization for face processing in ASD, as suggested by EEG studies [49–51].

No differences between groups were observed for the mouth and the outer facial features AOIs. These results are in contradiction with those of Chawarska and Shic [5] who reported that children with ASD spent less time looking at the mouth and outer facial features than TD children. A notable difference between their paradigm and the one used in the present study was the length of presentation of face stimuli (10 vs. 3.5 seconds respectively). Consistent with this idea, de Wit et al. [16], who also had a presentation time of 10 seconds, observed a marginal trend towards a decrease in the total fixation time of the mouth in children with ASD. Conversely and according to the result of the present study, Falck-Ytter et al. [17], who had a shorter presentation time (4 seconds), reported no difference between groups for the mouth.

In summary, with the exception of a difference observed in the right and the left eye, our study shows that during the first 3.5 seconds of presentation, visual scanning of faces is roughly similar between groups. Young children with ASD do not demonstrate general abnormalities in visual scanning of static faces, except for left/right distribution of gaze to the eyes. We nevertheless want to stress that the possibility of finding differences between groups likely depends on stimulus presentation duration. On this basis, we propose that children with ASD are initially subject to the same constraints for sampling different facial features, and that is only after this first comprehensive sampling (about 4 seconds) [14], that differences in scanning between groups may appear, reflecting strategic differences in visual processing [28]. A short stimulus presentation time (i.e. < 3 seconds) may in future studies also reveal differences in early visual sampling. All of these observations argue for a more detailed examination of the time course of visual scanning faces in future studies.

The second aim of the present study was to develop a new analytical approach based on transitions between AOIs. This new approach considers transition matrices as networks. We developed a measure of degree centrality for each AOI that quantifies its importance during visual scanning based on the number of AOIs to which it is directly connected, and on the total number of transitions associated with this specific AOI.

The results of this analysis indicate that the degree centrality in the left eye is lower for children with ASD compared with TD children. Although this result should be considered with caution and requires replication, this could nevertheless indicate that the left eye generates more exploratory activity among TD children. In a previous study [25], we demonstrated the presence of a bias towards the left hemifield for the first fixation in TD children but not in children with ASD. In addition, the first fixation on faces is generally directed toward the eye region [52]. Given these two observations, we can reasonably assume that the first fixations of TD children are mainly directed to the left eye region, when faces are presented at central vision [43]. Together these results suggest that the left eye region may be an anchor for the visual sampling of the different facial features in TD children. This hypothesis is further supported by the between-group comparison of correlation coefficients between fixation time and degree centrality of each AOI. The results indicate that the total fixation time on the left eye is differently related to the degree centrality in each group. In the group of children with ASD, the total fixation time is almost exclusively dependent on exploratory activity. In contrast, although exploratory activity explains a significant proportion of the variance of fixation time in TD, one or more other factors may also contribute to it. Using the measure of degree centrality in combination with that of the total fixation time in future studies should enable to better specify the visual scanning of faces by individuals with ASD.

The results obtained by the analysis of transition matrices illustrate the importance of developing new analytical measures that would enable the formulation of new working hypotheses [53]. Network analysis of transition matrices in other face perception tasks and in other tasks (e.g. social scene perception) will indicate how much new information can be generated from this approach. Furthermore, network analysis provides many more measures/conceptions of point centrality other than degree, such as betweenness (i.e. frequencies with which an AOI falls between pairs of other AOIs) that would certainly be worthwhile to apply in larger networks of AOIs.

The limitations of the network analysis approach are identical to those based on fixation time and involve the same problems as those associated with the use of AOIs [37]: results produced by these two approaches are notably influenced by the choices made by the researchers to define their AOIs (e.g. their size, shape). In contrast, the network analysis approach has the advantage to be less influenced by fixation identification thresholds settings and data quality than the approach based on fixation time, since network analysis does not take into account the duration of fixations [54–56].

## Conclusion

In conclusion, our data show that differences in visual scanning of faces between TD and ASD children are likely to be concentrated in the eye region. Furthermore, our study highlights the importance of developing innovative measures to perform eye-tracking data analyses, and underscores the importance of specifically examining exploratory behaviors and the microstructure of eye movements of individuals with ASD.

## Supporting Information

S1 Table Individual data underlying the findings reported in this study. (XLSX)
